# FEEding DURing red cell transfusion (FEEDUR RCT): a multi-arm randomised controlled trial

**DOI:** 10.1186/s12887-020-02233-3

**Published:** 2020-07-14

**Authors:** Tim Schindler, Kee Thai Yeo, Srinivas Bolisetty, Joanna Michalowski, Alvin Hock Kuan Tan, Kei Lui

**Affiliations:** 1 Department of Newborn Care, Royal Hospital for Women, Sydney, Australia; 2 School of Women’s and Children’s Health, University of New South Wales, Sydney, Australia; 3 Department of Neonatology, KK Women’s & Children’s Hospital, Singapore, Singapore; 4 Department of Neonatal and Perinatal Medicine, Flinders Medical Centre, Adelaide, Australia

**Keywords:** Preterm infant, Clinical trial, Near-infrared spectroscopy, Necrotizing enterocolitis

## Abstract

**Background:**

Necrotising Enterocolitis (NEC) is a devastating neonatal disease. A temporal association between red cell transfusion and NEC has been recognized and there have been concerns about the effects of feeding during transfusion. We aimed to assess the effect of different enteral feeding regimens on splanchnic oxygenation in preterm infants receiving red cell transfusions.

**Methods:**

This was an open, multi-arm, parallel-group, randomised controlled trial conducted in a single centre in Australia. We compared three different enteral feeding regimes during a single red cell transfusion in preterm infants < 35 weeks gestational age at birth. Infants were randomised to either: (1) Withholding enteral feeds for 12 h from the start of transfusion or; (2) Continuing enteral feeds or; (3) Restriction of enteral feed volume to 120 ml/kg/day (maximum 20 kcal/30 ml) for 12 h. The primary outcome was mean splanchnic-cerebral oxygenation ratio (SCOR) and mean splanchnic fractional oxygen extraction (FOE) before (1 h prior), during (1 h into transfusion) and after (end of transfusion; 12 and 24 h post) transfusion.

**Results:**

There were 60 transfusion episodes (20 transfusion episodes in each group) included in the analysis. 41 infants with a median gestational age at birth of 27 weeks (range 23–32 weeks) were enrolled. The median postnatal age was 43 days (range 19–94 days) and the median pre-transfusion haematocrit was 0.27 (range 0.22–0.32). All three groups were similar at baseline. There were no differences in mean SCOR and mean splanchnic FOE at any of the pre-specified time points. There were also no differences in clinical outcomes. There were no episodes of NEC in any infant. Across all groups the mean SCOR increased from the start to the end of each transfusion (0.97 [CI95% 0.96–0.98] vs 1.00 [CI95% 0.99–1.01]; *p* = 0.04) and the mean FOE decreased from the start to the end of each transfusion (0.22 [CI95% 0.21–0.23] vs 0.17 [CI95% 0.16–0.18]; *p* < 0.001).

**Conclusions:**

There were no differences in splanchnic oxygenation when enteral feeds were either withheld, continued or restricted during a transfusion. However, the successful conduct of this study supports the feasibility of a large trial powered to assess clinical outcomes.

**Trial registration:**

ANZCTR, ACTRN12616000160437. Registered 10 February 2016, https://www.anzctr.org.au/Trial/Registration/TrialReview.aspx?id=370069

## Background

Necrotising enterocolitis (NEC) is a serious inflammatory gut disease that affects 1 in 20 very preterm infants [1]. About 1 in 3 infants with NEC die or need surgery and many survivors have long-term health problems like poor growth and developmental delay [2]. The association between red blood cell (RBC) transfusions and the development of NEC in preterm infants has been increasingly recognised [3, 4]. This association between RBC transfusion and NEC may be coincidental as most preterm infants will receive transfusions within the first 4 weeks of life, which is the same time frame for the development of NEC. Further, it has been proposed that severe anaemia rather than the transfusion may be the precipitant event [5].

Mechanisms related to the development of transfusion-associated NEC (TANEC) are unknown. Several hypotheses have been proposed including the prolonged storage of blood, increased viscosity of blood, hypoperfusion-reperfusion injury and enteral feeding [5–8]. The effect of enteral feeding during RBC transfusion on splanchnic perfusion and oxygenation has not been fully elucidated. Theoretically, RBC transfusion may eliminate the usual increase in splanchnic blood flow that follows feeding, placing infants at risk of hypoperfusion. Withholding feeds may prevent this and reduce the risk of NEC [9]. Due to the association between NEC and feeding practices, there have been concerns about the effects of feeding during RBC transfusion [10–12]. As a result, there is a wide variety of feeding practices during RBC transfusion of preterm infants including withholding of feeds to reduce the risk of NEC.

Near-infrared spectroscopy (NIRS) allows for the non-invasive real-time measurement of tissue oxygenation [13]. Observational studies have suggested that feeding during RBC transfusion may alter splanchnic tissue oxygenation and predispose infants to the development of TANEC [9]. In this pilot study, we aimed to systematically study the effect of three different enteral feeding regimens on splanchnic oxygenation in preterm infants receiving RBC transfusions. We hypothesised that enteral feeding during RBC transfusions would have no effect on splanchnic oxygenation.

## Methods

### Trial design

We conducted a single-centre, open, parallel-group, randomised control trial, investigating three different enteral feeding regimes during a single RBC transfusion.

### Participants

Preterm infants admitted to the Neonatal Intensive Care Unit at the Royal Hospital for Women were enrolled between July 2016 and November 2017. Eligibility criteria for study inclusion were: Preterm infants born at < 35 weeks’ gestation; Receiving RBC transfusion for anaemia; Enteral feeding of at least 120 ml/kg/day. Exclusion criteria were: < 28 weeks’ corrected gestation at time of RBC transfusion; Growth restriction (birth weight < 3rd centile); Major congenital anomalies (including severe cardiac or cerebral disease, any malformation or disease of the gastrointestinal tract); Diagnosis of necrotising enterocolitis, spontaneous intestinal perforation or history of abdominal surgery; Need for vasopressor therapy at study entry point; Cutaneous disease not allowing for placement of NIRS sensor.

### Interventions

Infants were randomised to either: (1) Withholding enteral feeds for 12 h from the start of the transfusion or; (2) Continuing enteral feeds or; (3) Restriction of enteral feed volume to 120 ml/kg/day (maximum calorie concentration 20 kcal/30 ml) for 12 h from the start of the transfusion. Infants who did not receive enteral feeds during the transfusion were given intravenous fluids until enteral feeds were restarted. Infants who received enteral feeds during the transfusion continued according to the feeding regimen prior to the transfusion, which may have been continuous feeds or bolus feeds every 1–3 h. All transfusions were given over 4 h as per local policy. Each transfusion episode was treated as a separate event. Infants were assessed for eligibility and randomized with each transfusion.

### NIRS measurements

NIRS readings were obtained using the Nonin SenSmart Model X-100 Universal Oximetry System (NONIN Inc., Minnesota, USA), which was calibrated to continuously record. Neonatal sensors were secured with tape to the lateral aspect of the forehead to measure cerebral oxygenation and below the umbilicus to measure splanchnic oxygenation. Sensors were placed 1 h prior to the start of the transfusion and removed 24 h later. Vital signs including oxygen saturations (SaO2) were recorded at the same time as NIRS measurements.

NIRS data for extraction were identified by predetermined time stamps. NIRS data for each measurement contained approximately 150 data points (one data point every 4 s over 10 min). Artefacts as a result of infant movement or incorrect sensor position were noted and eliminated at the stage of data analysis. Splanchnic cerebral oxygenation ratio (SCOR), cerebral and splanchnic fractional oxygen extraction (cerebral FOE and splanchnic FOE) were determined from the average raw data of regional cerebral saturation (rSO_2_C) and regional splanchnic saturation (rSO_2_S), measured by NIRS. The following calculations were applied [14]:.

$$ \mathrm{SCOR}=\frac{\ \mathrm{rS}{\mathrm{O}}_2\mathrm{S}}{\ \mathrm{rS}{\mathrm{O}}_2\mathrm{C}} $$

$$ \mathrm{Cerebral}\ \mathrm{FOE}=\frac{\ \mathrm{Sa}{\mathrm{O}}_2-\mathrm{rS}{\mathrm{O}}_2\mathrm{C}}{\mathrm{Sa}{\mathrm{O}}_2} $$

$$ \mathrm{Splanchnic}\ \mathrm{FOE}=\frac{\ \mathrm{Sa}{\mathrm{O}}_2-\mathrm{rS}{\mathrm{O}}_2\mathrm{C}}{\mathrm{Sa}{\mathrm{O}}_2} $$

### Outcomes

#### Primary outcomes

1. Mean SCOR measurements over 4 time periods: pre-transfusion (1 h prior to transfusion), during transfusion (1 h into transfusion), end of transfusion, post-transfusion (12 and 24 h after end of transfusion).
2. Mean splanchnic FOE measurements.

#### Secondary outcomes (assessed by review of medical records)

1. Time to return to full feeds, defined as the number of hours after the 12 h feeding regimen until the infant is receiving the same feed volume as prior to the transfusion (at the discretion of the treating team).
2. Feed intolerance, defined by local guidelines at the time of the study (gastric aspirates > 30% of feed volume or vomiting).
3. Abdominal distension.
4. Adverse events including transfusion reactions, suspected or proven sepsis, TANEC (NEC within 48 h of transfusion).
5. Necrotising enterocolitis.
6. Late onset sepsis.
7. Mortality.

### Sample size

Based on the only available published reference values in preterm neonates at the time of study design [15], we expected mean regional splanchnic oxygen saturations to be approximately 70% with a standard deviation of approximately 7%. To detect a 10% change in mean splanchnic oxygenation, a sample size of at least 16 infants would be required to achieve 80% power at 0.05 level. We planned to recruit 20 infants per group.

### Randomisation and blinding

Infants were randomised using a random allocation sequence in a 1:1:1 ratio. Allocations were concealed in opaque, sealed envelopes. A non-clinical investigator was responsible for the random allocation sequence. Members of the clinical team enrolled and assigned participants.

This was an unblinded study, with feeding regimen assignments open to families, clinical staff and investigators.

### Statistical methods

All analyses were performed by intention-to-treat and all 60 transfusion episodes were included in analyses of all outcomes. Given normal distribution of data, we used parametric statistics for our analyses. We used paired t-tests to determine any difference in pre-transfusion mean SCOR and post-transfusion SCOR within groups. Independent t-tests were used to evaluate differences in mean SCOR between groups at all time points. We used repeated measures ANOVA to analyse changes in SCOR over the four pre-specified time points. Post-hoc tests, such as Tukey’s, were performed to further analyse significant results. When assessing differences between the three groups, we stated a priori that we expected that the time points where we expected to see significant differences were at the end of the transfusion (4 h post-starting) and at the point the feeding regimen returned to normal (12 h post-starting). We expected the observed differences to be most likely between infants in the full feeds group and no feeds group. The statistical analysis accommodated for infants who were enrolled in the study more than once for discrete transfusion episodes and for any infants with missing data points. Clinical outcome data were analysed using a combination of chi-squared, ANOVA and Kruskall-Wallis tests as appropriate.

## Results

**Fig. 1:**
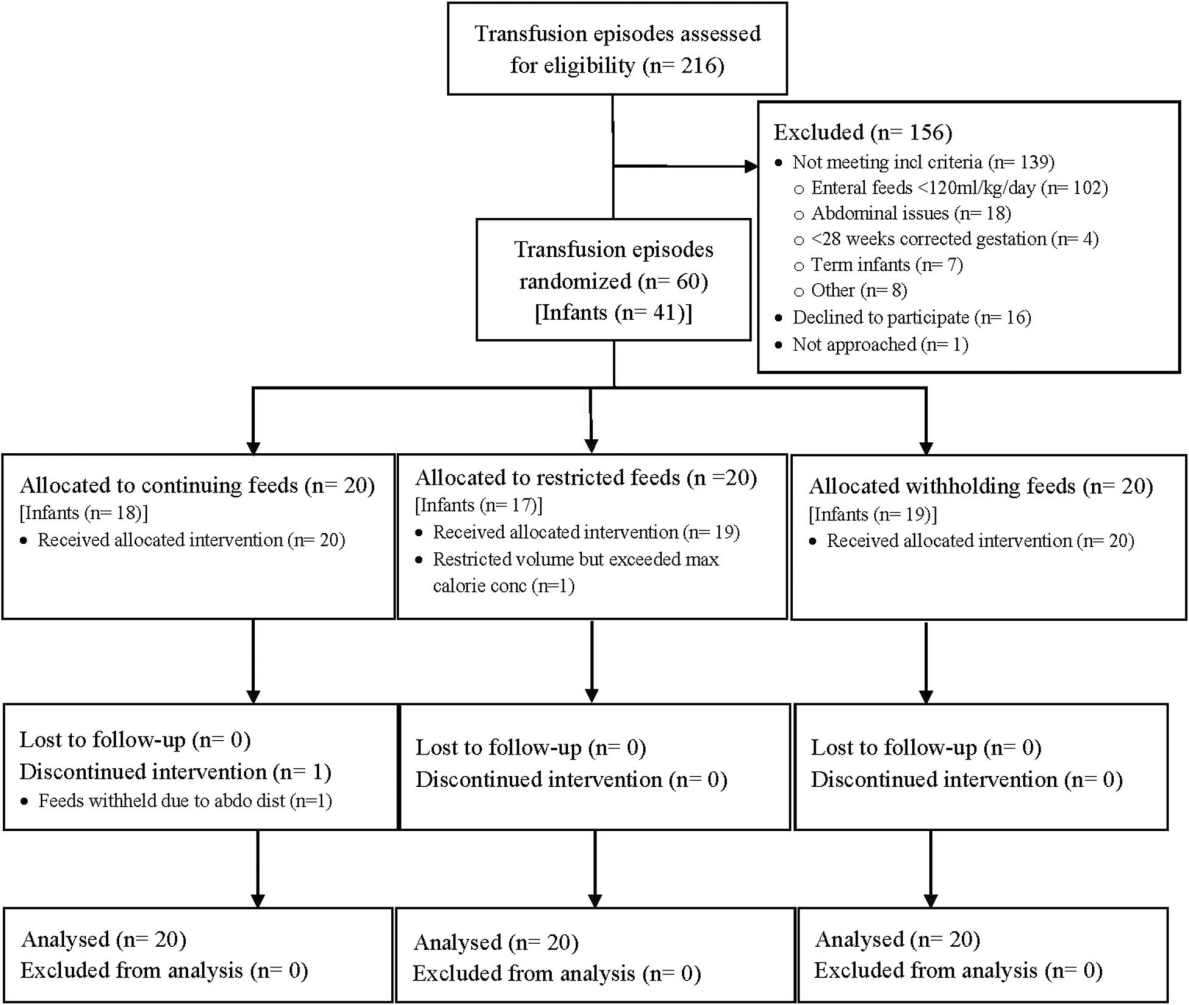
Trial flowchart. Consolidated Standards of Reporting Trials diagram for participant flow through the study

Figure 1 shows the Consolidated Standards of Reporting Trials diagram for recruitment. There were 60 transfusion episodes (20 transfusion episodes in each group) included in the analysis. One infant allocated to restricted feeds was not restricted to a maximum calorie concentration of 20 kcal/30 mL. One infant allocated to continuing feeds discontinued feeds due to abdominal distension during the observation period.

Table 1 shows the baseline demographic and clinical characteristics for each group. 41 infants with a median gestational age at birth of 27 weeks (range 23–32 weeks) were enrolled. All three groups had similar perinatal characteristics. Infants allocated to restricted feeds had a lower corrected gestational age at the time of transfusion.

**Table 1:** Perinatal Characteristics

	Group 1 No feeds *n* = 19	Group 2 Full feeds *n* = 18	Group 3 Restricted *n* = 17	*p*-value
Gestation at birth, mean ± SD	27.2 ± 2.3	26.4 ± 2.1	26.4 ± 1.9	0.38
Birth weight, mean ± SD	1005 ± 331	847 ± 218	903 ± 214	0.16
Birth weight percentile, mean ± SD	52 ± 26	49 ± 29	60 ± 30	0.46
Female sex, n (%)	10 (50%)	11 (55%)	11 (55%)	0.94
Singleton birth, n (%)	18 (90%)	17 (85%)	18 (90%)	0.85
Caesarean delivery, n (%)	14 (70%)	12 (60%)	10 (50%)	0.44
Maternal age, mean ± SD	32 ± 5	32 ± 5	31 ± 4	0.88
Received antenatal corticosteroids, n (%)	20 (100%)	20 (100%)	20 (100%)	n/a
Received antenatal magnesium, n (%)	11 (55%)	17 (85%)	13 (65%)	0.12
Grade III/IV intraventricular haemorrhage, n (%)	0	0	1 (5%)	0.36
Early onset sepsis, n (%)	0	0	1 (5%)	0.36
Postnatal age, mean ± SD	46 ± 15	48 ± 18	39 ± 14	0.14
Corrected gestational age, mean ± SD	34.4 ± 4.0	33.3 ± 1.9	31.8 ± 2.3	0.02*
Weight at time of study, mean ± SD	1702 ± 640	1512 ± 524	1440 ± 500	0.32
Haemoglobin pre-transfusion, mean ± SD	93 ± 8	92 ± 8	94 ± 7	0.66
Haematocrit pre-transfusion, mean ± SD	0.27 ± 0.02	0.27 ± 0.03	0.28 ± 0.02	0.72
Invasive ventilation at time of study, n (%)	0	3 (15%)	1 (5%)	0.15
Non-invasive respiratory support at time of study, n (%)	16 (80%)	14 (70%)	18 (90%)	0.29
Inspired oxygen at start of study period, mean ± SD	0.26 ± 0.08	0.28 ± 0.10	0.28 ± 0.08	0.65
Transfusion episodes (n)**	20	20	20	

*p < 0.05

**Each transfusion episode treated as a discrete event

**Fig. 2:**
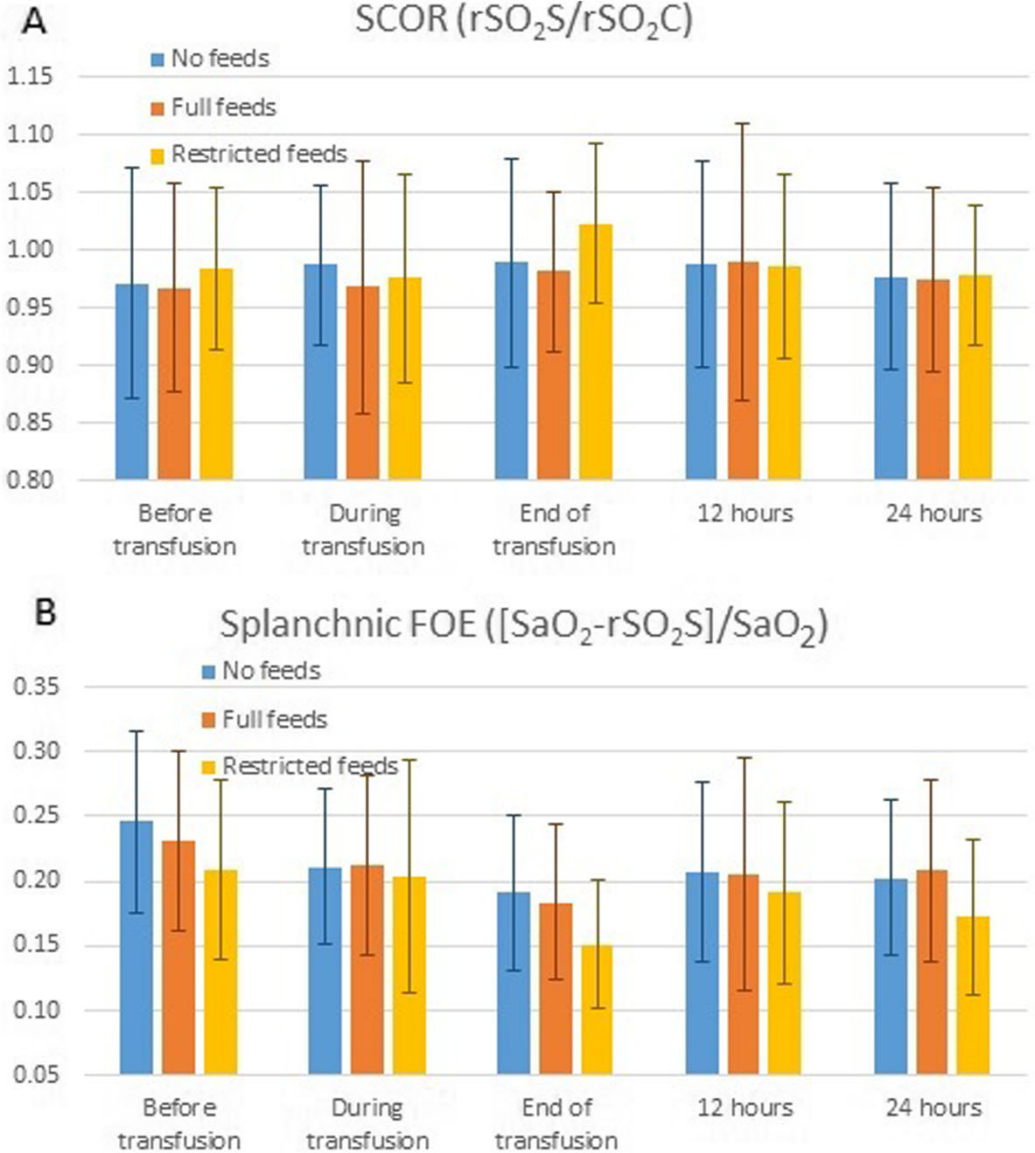
Splanchnic oxygenation at pre-specified time points. **a** Mean (SD) splanchnic cerebral oxygenation ratio (SCOR; rSO2S/rSO2C); **b** Mean (SD) splanchnic fractional oxygen extraction (FOE; [SaO2-rSO2S]/SaO2)

There were no differences in mean SCOR and mean splanchnic FOE at any of the pre-specified time points (see Fig. 2; Table 2). Before transfusion, there were no differences between groups (mean SCOR No feeds – 0.97 ± 0.10 vs Full feeds – 0.97 ± 0.09 vs Restricted feeds 0.98 ± 0.07 [*p* = 0.72]; mean FOE No feeds – 0.25 ± 0.07 vs Full feeds – 0.22 ± 0.07 vs Restricted feeds 0.20 ± 0.07 [*p =* 0.72]). Similarly, there were no differences between groups at the end of the transfusion (mean SCOR No feeds – 0.99 ± 0.09 vs Full feeds – 0.98 ± 0.07 vs Restricted feeds 1.02 ± 0.07 [*p* = 0.20]; mean FOE No feeds – 0.19 ± 0.06 vs Full feeds – 0.18 ± 0.06 vs Restricted feeds 0.16 ± 0.05 [*p* = 0.16]) or 12 h after starting the transfusion when the feeding regime returned to normal (mean SCOR No feeds – 0.99 ± 0.09 vs Full feeds – 0.99 ± 0.12 vs Restricted feeds 0.99 ± 0.08 [*p* = 1.0]; mean FOE No feeds – 0.21 ± 0.07 vs Full feeds – 0.19 ± 0.09 vs Restricted feeds 0.20 ± 0.07 [*p* = 0.83]).

**Table 2:** NIRS values for splanchnic oxygenation at pre-specified time points

Splanchnic saturations – mean (SD)					
Time point	Before transfusion	During transfusion	End of transfusion	12 h	24 h
No feeds	74 (7.2)	76 (5.3)	78 (5.2)	77 (6.0)	77 (5.4)
Full feeds	75 (7.1)	76 (7.4)	79 (6.0)	77 (8.9)	76 (6.9)
Restricted feeds	76 (5.6)	76 (7.4)	80 (4.3)	77 (5.9)	77 (4.9)
*p*-value	0.60	0.98	0.40	0.96	0.81
SCOR – mean (SD)					
Time point	Before transfusion	During transfusion	End of transfusion	12 h	24 h
No feeds	0.97 (0.10)	0.99 (0.07)	0.99 (0.09)	0.99 (0.09)	0.98 (0.09)
Full feeds	0.97 (0.09)	0.97 (0.11)	0.98 (0.07)	0.99 (0.12)	0.98 (0.08)
Restricted feeds	0.98 (0.07)	0.98 (0.09)	1.02 (0.07)	0.99 (0.08)	0.98 (0.06)
*p*-value	0.72	0.74	0.20	1.0	0.99
Mesenteric FOE – mean (SD)					
Time point	Before transfusion	During transfusion	End of transfusion	12 h	24 h
No feeds	0.25 (0.07)	0.21 (0.06)	0.19 (0.06)	0.21 (0.07)	0.20 (0.06)
Full feeds	0.23 (0.07)	0.21 (0.07)	0.18 (0.06)	0.20 (0.09)	0.21 (0.07)
Restricted feeds	0.21 (0.07)	0.20 (0.09)	0.15 (0.05)	0.19 (0.07)	0.17 (0.06)
*p*-value	0.21	0.86	0.16	0.83	0.41

With respect to clinical outcomes, there were no differences in: Time to return to full feeds; Abdominal distension; Adverse events; TANEC (see Table 3). One infant in the restricted feeds arm was treated for suspected NEC, which was not subsequently proven. There were no episodes of proven NEC or mortality in any infant involved in the study. Feed intolerance (predominantly due to gastric aspirates > 30% of feed volume) occurred more frequently in infants in the full feeds arm (No feeds 0% vs Full feeds 25% vs Restricted feeds 5%; *p* = 0.02).

**Table 3:** Clinical Outcomes. NEC – Necrotising Enterocolitis; TANEC – Transfusion-Associated NEC (NEC within 48 h of transfusion)

	Group 1 No feeds	Group 2 Full feeds	Group 3 Restricted feeds	*p*-value
Time to return to full feeds, hours ± SD	8 ± 35	13 ± 46	8 ± 32	0.88
Feed intolerance, n (%)	0	5 (25%)	1 (5%)	0.02*
Abdo distension, n (%)	1 (5%)	3 (15%)	2 (10%)	0.57
Adverse Events, n (%)	0	0	1 (5%)**	0.36
TANEC, n	0	0	0	n/a
NEC, n	0	0	0	n/a***
Late onset sepsis, n	11/19	10/18	7/17	n/a***
Mortality, n	0	0	0	n/a***

*p < 0.05

**1 infant treated for suspected NEC (managed conservatively; not proven)

***not appropriate to compare rates of long term outcomes as feeding allocation was for one transfusion only

Across all groups, regional splanchnic saturations increased from the start to end of each transfusion (75 ± 6.6 vs 79 ± 5.2; MD 4.3 [95% CI 2.5–6.0]). The mean SCOR increased from the start to end of each transfusion (0.97 ± 0.09 vs 1.00 ± 0.08; MD 0.03 [95% CI 0.01–0.05]) and the mean FOE decreased from the start to end of each transfusion (0.22 ± 0.07 vs 0.17 ± 0.06; MD -0.05 [95% CI -0.03 – − 0.07]).

## Discussion

The results of this study demonstrated no differences in splanchnic oxygenation between feeding regimens either during transfusion or at any time point up to 24 h after transfusion. This was despite a consistent increase in regional splanchnic saturations from the start to the end of the transfusion in all three groups. These findings are in keeping with the hypothesis that there is an opportunity for ischaemic damage to the gastrointestinal system prior to a blood transfusion, which is followed by rapid reperfusion and an inflammatory cascade during and immediately after the transfusion [16]. However, this study found that changing the feeding regimen during transfusion had no effect on these physiological changes. This is not surprising given emerging evidence that severe anaemia, as opposed to the transfusion itself, may be the sentinel event that predisposes preterm infants to the development of NEC [5].

All studies that utilise NIRS technology are limited by a lack of standardisation with respect to processing and interpreting output data. In this study, we elected to average NIRS values over ten minutes at each pre-specified time point but it is currently unclear whether this is optimal. Similarly, differences in variability or time below a threshold value may be a more appropriate representation of splanchnic blood flow compared with an average over time. NIRS data were extracted at five pragmatically selected time points, potentially missing differences in splanchnic oxygenation at other times during the observation period. In addition, we did not control for timing of feeding nor investigate response to feeding, which may have added value to the study. Splanchnic blood flow is normally increased in response to enteral feeds but RBC transfusion has been shown to suppresses this postprandial increase in preterm infants [9, 17, 18]. This postprandial increase is also absent in infants who develop NEC [19].

With regard to applicability in clinical practice, it is difficult to know how far we can extrapolate findings from physiological studies such as this. Splanchnic NIRS measurements have not been validated in the same way as cerebral NIRS measurements [20] and this fundamentally limits what we can infer clinically from these results. The study was limited by a low background incidence of NEC, with no eligible infant developing NEC during the study period or at any stage during hospitalisation. It is also important to acknowledge that we excluded infants less than 28 weeks’ corrected gestation at the time of RBC transfusion due to individual clinician concerns. It is possible that differences in splanchnic oxygenation would have been apparent in these high risk infants. Further, each transfusion episode was treated as a separate event. Although this choice suited the primary outcomes of the study, it places significant limitations on the interpretation of secondary clinical outcomes. We also acknowledge that there is significant clinical practice variation with respect to the timing of withholding feeds around transfusion in units that have adopted this strategy.

The absence of demonstrable differences in splanchnic oxygenation between feeding regimens highlights the importance of conducting a trial that investigates clinical outcomes. The effect of feeding a baby during a RBC transfusion and the subsequent development of NEC remains unclear [21]. A systematic review of “before-after” studies (7 studies; 7492 infants) showed a significant reduction in the risk of developing NEC within 48–72 h when feeds were withheld during a transfusion [22]. This should be interpreted with caution as historical comparisons are at high risk of ascertainment bias and regression to the mean [23]. Consequently, there are a variety of opinions about the optimal feeding regime for a baby having a RBC transfusion and there is consequently significant practice variation [24, 25]. The prevention of NEC has been identified as a top research priority in the care of preterm infants yet this question remains unanswered [26].

## Conclusions

Inherently limited by a low background incidence of NEC, this study did not demonstrate any physiological differences in splanchnic oxygenation when enteral feeds were either withheld, continued or restricted during a transfusion. However, the successful conduct of this study suggests that a large trial powered to assess clinical outcomes is achievable to answer this important clinical question.

## Data Availability

All data generated or analysed during this study are included in this published article.

## Abbreviations

FEEDUR RCT: FEEding DURing Red Cell Transfusion
NEC: Necrotising Enterocolitis
SCOR: Splanchnic-Cerebral Oxygenation Ratio
FOE: Fractional Oxygen Extraction
RBC: Red Blood Cell
TANEC: Transfusion-Associated Necrotising Enterocolitis
NIRS: Near-infrared Spectroscopy
rSO_2_C: Regional Cerebral Saturation
rSO_2_S: Regional Splanchnic Saturation
